# Local Anæsthesia

**Published:** 1866-08

**Authors:** 


### LOCAL ANÆSTHESIA.

Among the late novelties in surgical and medical appliances perhaps none has attracted more attention in these countries, during the past few months, than that of Dr. RICHARDSON, of London, for producing local anæsthesia by means of the spray of ether thrown upon the part. A considerable number of trials of this method of producing insensibility to pain during minor surgical operations have been made in this city. MR. SYMES, one of the surgeons to Stevens' Hospital has tried Dr. RICHARDSON'S plan with almost universal success. It has also been tried in the Meath Hospital, and reported upon favorably by the surgeons of that institution. MR. McCLEAN, the surgeon-dentist of the City of Dublin Hospital, has made use of this means of producing anæsthesia during the removal of teeth, and with a very satisfactory result.—*Med. & Sur. Rep.*

